# Highly Stable Nanocontainer of APTES-Anchored Layered Titanate Nanosheet for Reliable Protection/Recovery of Nucleic Acid

**DOI:** 10.1038/srep21993

**Published:** 2016-02-24

**Authors:** Tae Woo Kim, In Young Kim, Dae-Hwan Park, Jin-Ho Choy, Seong-Ju Hwang

**Affiliations:** 1 Department of Chemistry and Nanoscience, Ewha Womans University, Seoul 03760, Korea

## Abstract

A universal technology for the encapsulative protection of unstable anionic species by highly stable layered metal oxide has been developed via the surface modification of a metal oxide nanosheet. The surface anchoring of (3-aminopropyl)triethoxysilane (APTES) on exfoliated titanate nanosheet yields a novel cationic metal oxide nanosheet, which can be universally used for the hybridization with various biological and inorganic anions. The encapsulation of deoxyribonucleic acid (DNA) in the cationic APTES-anchored titanate lattice makes possible the reliable long-term protection of DNA against enzymatic, chemical, and UV−vis light corrosions. The encapsulated DNA can be easily released from the titanate lattice via sonication, underscoring the functionality of the cationic APTES-anchored titanate nanosheet as a stable nanocontainer for DNA. The APTES-anchored titanate nanosheet can be also used as an efficient CO_2_ adsorbent and a versatile host material for various inorganic anions like polyoxometalates, leading to the synthesis of novel intercalative nanohybrids with unexplored properties and useful functionalities.

Graphene, exfoliated two-dimensional (2D) monolayers of graphite, attracts a great deal of research interest because of its unique physicochemical properties of high electrical and thermal conductivity, great mechanical strength, and high flexibility as well as its useful functionalities as electrodes, catalysts, and nanobio materials^1^,^2^,^3^. Beyond graphene, recent research regarding 2D nanostructured materials focuses on the inorganic analogues of graphene, i.e. the exfoliated nanosheets of transition metal dichalcogenide, layered metal oxide, and layered metal double hydroxide (LDH)^4^,^5^,^6^. Among them, the exfoliated 2D nanosheets of layered metal oxides possess distinct negative surface charges originating from their inherent stoichiometry, which renders these materials useful building blocks for accommodating diverse cationic species^5^,^6^,^7^,^8^,^9^,^10^,^11^,^12^. For example, the intercalative hybridization of cationic LDH nanosheets with anionic titanate nanosheets leads not only to the formation of heterolayered LDH−titanate nanohybrid with efficient visible light photocatalytic activity but also to the remarkable improvement of the chemical stability of basic LDH nanosheet against acidic corrosion^12^. Like the LDH nanosheet, deoxyribonucleic acid (DNA), one of the most important biomolecules, suffers from inherently poor chemical and physical stabilities, which frustrate the universal commercialization of DNA-based genetic engineering technologies including cloning, polymerase chain reaction (PCR), recombinant DNA technology, DNA fingerprinting, gene therapy, DNA barcoding, and DNA profiling^13^,^14^. Since metal oxides generally show high chemical and thermal stability^12^,^13^,^14^, layered metal oxide nanosheets can be good candidates as robust nanocontainers for DNA. Despite the high stability of the exfoliated metal oxide nanosheet, its negative surface charge makes it unsuitable for hybridization with anionic species like DNA^7^,^8^,^9^,^10^,^11^,^12^. A fundamental way to overcome the inherent limitation of the layered metal oxide nanosheet is to change its negative surface charge to a positive one^15^. The resulting surface-modified cationic metal oxide nanosheet can be used as a host material for accommodating a wide spectrum of anionic guest species. In addition to biomolecular anions like DNA, there are many other candidate anions for hybridization with the cationic metal oxide nanosheet, leading to the resulting novel heterostructured nanohybrids. In one instance, polyoxometalate clusters serving as the polyatomic inorganic anions showed versatile functionalities in electrodes, electrocatalysts, photocatalysts, and redox catalysts^16^,^17^,^18^. The resulting nanohybrid materials composed of polyoxometalate and layered metal oxide may show many novel functions via the synergistic coupling of these two components. At the time of the submission of this study, we are aware of no report on the hybridization of cationic layered metal oxide nanosheet with diverse anionic species including DNA and inorganic nanoclusters as well as the application of the obtained metal oxide nanosheets as DNA reservoir and CO_2_ adsorbent.

Here we report the synthesis of cationic layered titanate nanosheet via the anchoring of (3-aminopropyl)triethoxysilane molecules (APTES) on the surface of exfoliated layered titanate and the following exfoliation by the fine-tuning of the pH of suspension. This cationic nanosheet can act as a unique storage matrix efficient for the preservation of DNA under corrosive environments (e.g., digestion with the DNase I, or low pH solution and UV−vis irradiation, or long-term storage) as well as for the accommodation of diverse polyoxometalate anions in its interlayer space. Also very promising adsorption of CO_2_ gas can be achieved with this APTES-anchored titanate nanosheet.

## Results

### Cationic APTES-Anchored Titanate Nanosheet

**Figure 1:**
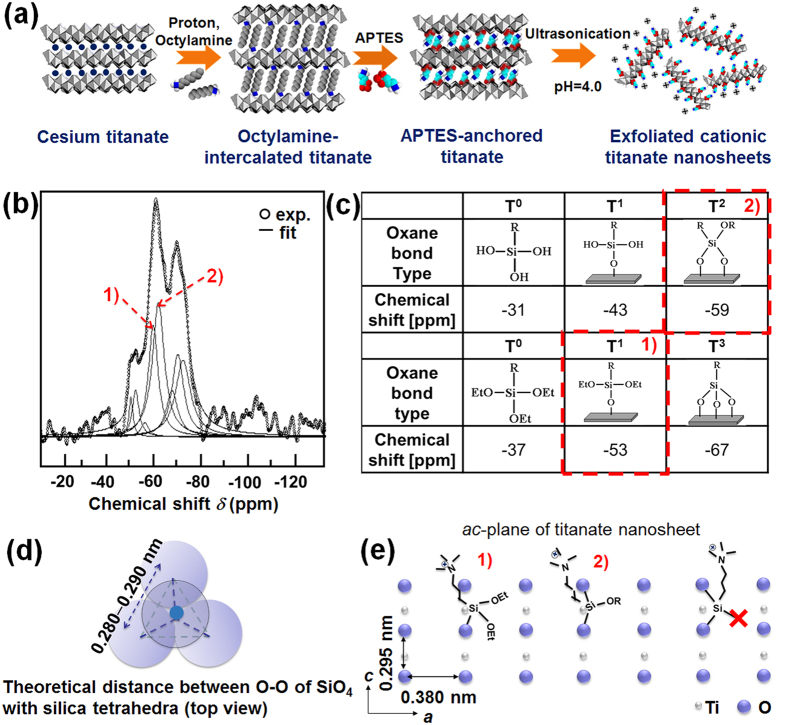
Surface modification of layered titanate with APTES. (**a**) A schematic model for the synthesis of APTES-anchored layered titanate nanosheet. (**b**) ^29^Si MAS-NMR spectrum of APTES-anchored layered titanate. (**c**) Schematic models and chemical shifts for several oxane bonds. (**d**) Top view of the molecular model of tetrahedral SiO_4_. (**e**) Schematic model for the anchoring of APTES molecule on the *ac*-plane of layered titanate.

As illustrated in Fig. 1a, the surface modification of layered titanate is achieved by the anchoring of APTES on the surface of the titanate lattice. Prior to the anchoring of APTES, the interlayer cesium ions in the pristine titanate (Cs_0.67_Ti_1.83_O_4_) are exchanged with protons, and then octylamine and APTES are sequentially intercalated into the titanate lattice. Since the neutral amine group of APTES is spontaneously protonated to cationic ammonium group at low pH, the exfoliation of APTES-anchored layered titanate layers can occur by lowering the pH due to an electrostatic repulsion between ammonium groups of adjacent titanate layers. The intercalation of octylamine and APTES in the titanate lattice are confirmed by powder X-ray diffraction (XRD) and Fourier transformed-infrared (FT-IR) spectroscopic analyses. According to powder XRD analysis, the gallery heights of the octylamine- and APTES-intercalated layered titanates are well matched with the molecular lengths of octylamine and APTES, respectively, clearly demonstrating the successful intercalation of octylamine and APTES into the titanate (Ti_1.83_O_4_^0.67−^) lattice (see Supplementary Information, Fig. S1)^19^. The intercalation of the octylamine and APTES on the layered titanate is further confirmed by the FT-IR results (see Supplementary Information, Fig. S2). The characteristic absorption bands of octylamine appear in the FT-IR spectrum of octylamine-intercalated layered titanate, confirming the stabilization of octylamine in the layered titanate lattice. The APTES-anchored titanate displays intense IR absorption bands at 990–1100 cm^−1^ and 1212 cm^−1^ corresponding to the stretching vibration of SiO–C and bending vibration of OC–H respectively, clearly demonstrating the successful exchange of octylamine with APTES in the titanate lattice^19^,^20^,^21^. Of prime importance is that an absorption band corresponding to Si–O–Ti bonds is discernible at ~970 cm^−1^  22,23 underscoring the anchoring of APTES molecules on the interlayer surface of layered titanate^21^,^22^,^23^.

The nature of the chemical bonding of APTES-anchored titanate is examined by ^29^Si magic angle spinning-nuclear magnetic resonance (MAS-NMR) spectroscopic analysis, as illustrated in Fig. 1b,c. The APTES-anchored titanate exhibits chemical shifts at −53 ppm and −59 ppm, indicating the formation of monodentate (T^1^)- and bidentate(T^2^)-type siloxane bonding between the layered titanate and APTES^19^. As depicted in Fig. 1d,e, the (O–O) bond distances of 0.280–0.285 nm in SiO_4_ tetrahedra are fairly similar to the *c*-axis lattice parameter of 0.290 nm in the layered titanate but much shorter than the *a*–axis lattice parameter of 0.380 nm^24^. Thus, the silicon atom in APTES molecule can bind with one or two oxygen atoms on the titanate layer aligned along the *c*-axis, leading to the formation of monodentate (T^1^)- or bidentate (T^2^)-type siloxane bonding. Conversely, the APTES molecule cannot form a tridentate (T^3^)-type siloxane bond with three oxygen atoms because of severe geometric mismatch between (O–O) bonds in tetrahedral SiO_4_ and those in the *ac*-plane of layered titanate. On the basis of the results of atomic emission spectrometry–inductively coupled plasma (AES–ICP) spectrometry, CHN elemental analysis, and thermogravimetric (TG) analysis, the chemical formulas of the octylamine-intercalated and APTES-anchored layered titanates are determined as (CH_3_(CH_2_)_7_NH_2_)_0.61_H_0.67_Ti_1.83_O_4_·1.5H_2_O and (APTES)_0.71_Ti_1.83_O_4_·0.8H_2_O, respectively.

**Figure 2:**
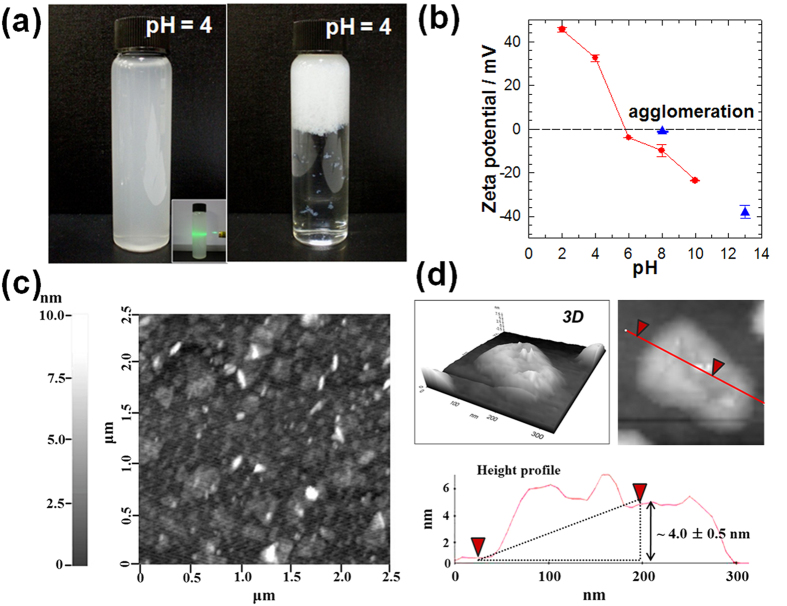
Exfoliation to cationic APTES-anchored titanate nanosheet. (**a**) Photographs of the colloidal suspensions of (left) APTES-anchored layered titanate nanosheet and (right) unmodified layered titanate nanosheet at pH = 4.0. (**b**) Zeta potential data of APTES-anchored layered titanate nanosheet (red) and unmodified layered titanate nanosheet (blue). (**c**) AFM image and (**d**) height profile of the APTES-anchored layered titanate nanosheet. Inset of (**a**) shows Tyndall phenomenon of the colloidal suspension of the APTES-anchored layered titanate nanosheet under the irradiation of light.

As presented in Fig. 2a, the resulting colloidal suspension of the APTES-anchored layered titanate nanosheet displays the distinct Tyndall phenomenon with good dispersion ability, underscoring the effective exfoliation of cationic titanate nanosheets^25^. Conversely unmodified layered titanate nanosheets rapidly agglomerate at pH 4.0, which is ascribable to significant electrostatic attraction between negatively charged titanate nanosheets and protons. The surface charge of the APTES-modified titanate nanosheet is quantitatively examined with a zeta potential analyzer, in comparison with that of the unmodified titanate nanosheet. As presented in Fig. 2b, the agglomeration of the unmodified titanate nanosheet begins to occur at pH 8.0, whereas the APTES-anchored layered titanate nanosheet shows a distinct positive zeta potential value at the pH less than 6.0 without agglomeration. This experimental finding clearly demonstrates the effective alteration of the negative charge of layered titanate nanosheet to positive charge by the anchoring of APTES. As can be seen clearly from Fig. 2c,d, the atomic force microscopic (AFM) analysis provides strong evidence for the formation of the 2D nanosheet of APTES-anchored layered titanate with the thickness of 4.0 nm ± 0.5 nm and lateral dimension of a few hundreds of nanometers. The thickness of the APTES-anchored titanate nanosheet is notably larger than the unmodified titanate monolayer, reflecting the immobilization of anchored APTES groups and/or the formation of hydration layers on the surface of nanosheets^25^,^26^.

### Hybridization of Cationic APTES-Anchored Titanate Nanosheet with DNA

**Figure 3:**
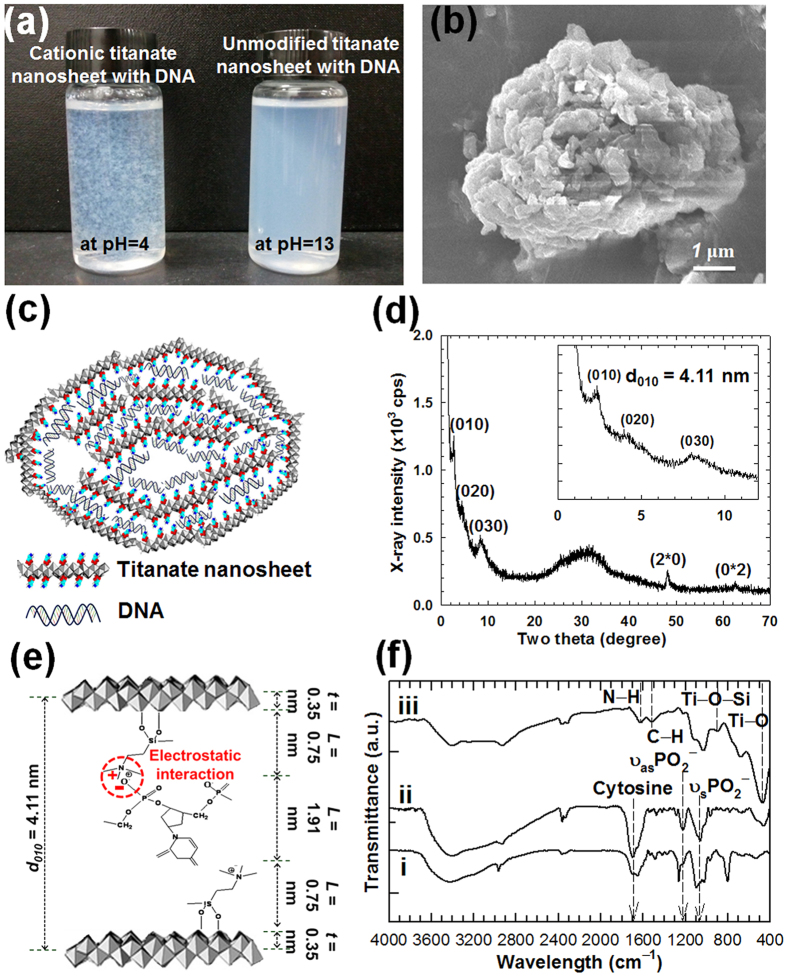
Hybridization of cationic APTES-anchored titanate nanosheet with DNA. (**a**) Photographs of the colloidal mixtures of (left) DNA and APTES-anchored layered titanate nanosheet and (right) DNA and unmodified layered titanate nanosheets. (**b**) FE-SEM image and (**c**) schematic model of the DNA–layered titanate nanohybrid. (**d**) Powder XRD pattern and (**e**) structural model of the DNA–layered titanate nanohybrid. (**f**) FT-IR spectra of i) precursor DNA, ii) the DNA–layered titanate nanohybrid, and iii) APTES-anchored layered titanate. Inset of (**d**) is expanded view in the region of 0–12°.

An electrostatic interaction between cationic APTES-anchored titanate nanosheets and anionic DNA gives rise to the immediate formation of DNA–layered titanate nanohybrid at pH 4.0, as presented in Fig. 3a. This sharply contrasts with no reaction of DNA with unmodified titanate nanosheets at pH 13.0. The present results clearly demonstrate a critical role of surface modification in the hybridization between titanate nanosheet and DNA.

According to the field emission-scanning electron microscopy (FE-SEM) and high resolution-transmission electron microscopy (HR-TEM) analyses, the DNA–layered titanate nanohybrid displays irregular spherical particles whose surface is wrapped by the titanate nanosheets, underscoring the encapsulation of anionic DNA molecules by the cationic nanosheets, see Fig. 3b,c, and Fig. S3. The crystal structure of the obtained DNA–layered titanate nanohybrid is examined by powder XRD analysis. The XRD pattern of the DNA–layered titanate nanohybrid shows an expanded gallery height of 1.91 nm, which is compatible with the diameter of DNA double helix (2.0 nm), see Fig. 3d,e 27,28,29. This result provides strong evidence for the intercalation of DNA molecules in the APTES-anchored layered titanate nanosheets. The observation of the in-plane diffraction patterns of (2*0) and (0*2) from XRD and selected area electron diffraction (SAED) data clearly demonstrates the maintenance of the 2D lattice of the titanate nanosheets upon the hybridization with DNA (see Fig. 3d and Fig. S3 of Supplementary Information)^30^. The encapsulation of DNA in the DNA–layered titanate nanohybrid is further confirmed by FT-IR analysis showing the characteristic IR bands of the phosphate symmetric and asymmetric stretching vibrations of DNA molecule at 1086 cm^−1^ and 1230 cm^−1^, respectively, as well as the Ti–O vibration at 474 cm^−1^ (Fig. 3f)^31^. According to EDS analysis, the present DNA–layered titanate nanohybrid can store 9.3 wt% of DNA (see Supplementary Information, Fig. S4). Although there are several reports regarding the hybridization of DNA with graphite and graphene, no quantitative characterization for the content of DNA hybridized was made for these graphite/graphene-based nanohybrids^32^,^33^,^34^. Instead, the SiO_2_ nanoparticle-based hybrid system was reported to show the DNA storage capability of 2.5 wt%^13^, which is notably smaller than that of the present DNA–layered titanate nanohybrid. This result strongly suggests the merit of 2D nanosheet morphology for accommodating large amount of biomolecules^5^.

### Role of Cationic Titanate Nanosheet as Nanocontainer for DNA

**Figure 4:**
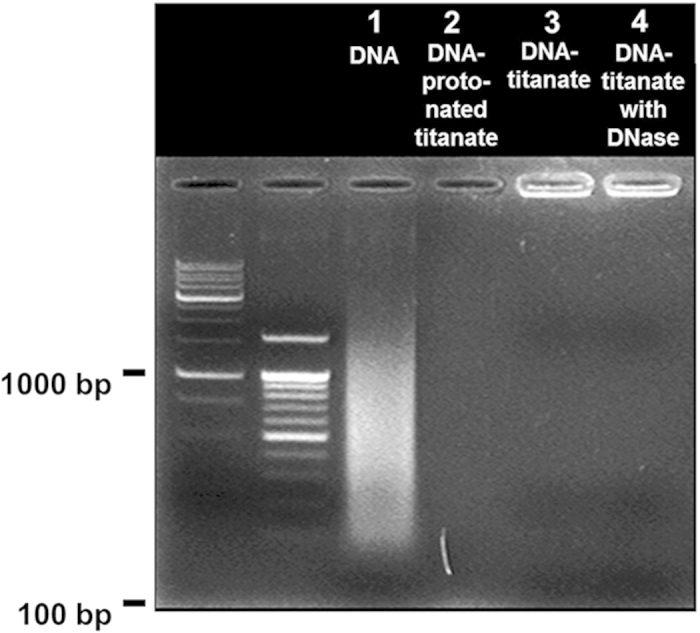
The stability of the DNA−layered titanate nanohybrid against enzymatic corrosion. Agarose gel-electrophoresis analyses for the DNA–layered titanate nanohybrids before and after the treatment of DNase I. Lane 1: the precursor DNA. Lane 2: the unmodified protonated titanate reacted with DNA. Lane 3: the DNA–layered titanate nanohybrid. Lane 4: the DNA–layered titanate nanohybrid digested with DNase I. The first and second lanes in the electrophoresis analysis are 1000 and 100 base-pair ladder DNA, respectively. The precursor DNA is used as a positive control.

The functionality of the cationic APTES-anchored titanate nanosheet as a stable nanocontainer for DNA is investigated by probing the effect of DNase I treatment on the stability of DNA encapsulated in the DNA–layered titanate nanohybrid. As presented in Fig. 4, the precursor DNA used for the hybridization with the cationic titanate nanosheet exhibits a wide spectrum of molecular size (lane 1). No signal of DNA is observed for the unmodified protonated titanate reacted with DNA (lane 2), underscoring no immobilization of DNA without surface modification. Conversely, a strong DNA signal concentrated on the cathode is discernible for the DNA–layered titanate nanohybrid (lane 3). No observation of the trail of DNA under electrical potential demonstrates the complete stabilization of various sized DNA molecules in the lattice of the DNA–layered titanate nanohybrid. Of prime importance is that the treatment with DNase I does not cause any trail of released DNA (lane 4), underscoring that the encapsulated DNA is protected by the cationic titanate nanosheets against an enzymatic corrosion.

**Figure 5:**
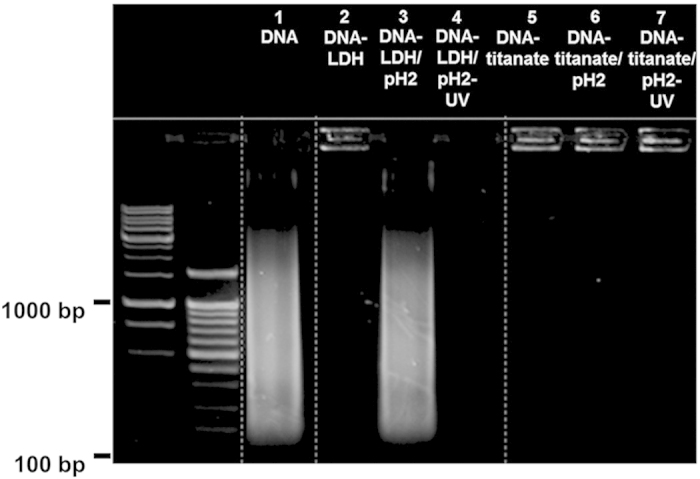
The stability of the DNA−layered titanate nanohybrid against acidic digestion and UV-vis illumination. Agarose gel-electrophoresis analyses for the DNA–LDH and DNA–layered titanate nanohybrids subjected to the acidic digestion and UV–vis illumination. Lane 1: the precursor DNA. Lane 2: the DNA–LDH nanohybrid. Lane 3: the acid-digested DNA–LDH nanohybrid. Lane 4: the acid-digested/UV–vis-irradiated DNA–LDH nanohybrid. Lane 5: the DNA–layered titanate nanohybrid. Lane 6: the acid-digested DNA–layered titanate nanohybrid. Lane 7: the acid-digested/UV–vis-irradiated DNA–layered titanate nanohybrid. The first and second lanes in the electrophoresis analysis are 1000 and 100 base-pair ladder DNA, respectively. The precursor DNA is used as a positive control.

The stability of the DNA–layered titanate nanohybrid is further tested with acidic corrosion and UV–vis irradiation, in comparison with that of the reference DNA–LDH nanohybrid. As depicted in Fig. 5, the DNA–layered titanate nanohybrid retains a DNA signal on the cathode without any release of DNA under acidic corrosion with UV–vis irradiation (lanes 6 and 7)^35^,^36^. The observed protection of DNA in the DNA–layered titanate nanohybrid against acidic etching and UV–vis irradiation is attributable to the excellent chemical stability and high UV absorption coefficient of the outer shell of layered titanate nanosheets. These characteristics of the layered titanate prevent the damage of encapsulated DNA by proton corrosion and UV-absorption (λ = 266 nm), respectively (see Supplementary Information, Fig. S5)^37^. Conversely, the acidic treatment for the DNA–LDH nanohybrid causes the immediate release of DNA within a few minutes via the dissolution of LDH lattice (lane 3)^35^,^38^. The acid-digested DNA–LDH nanohybrid displays a long trail of DNA signal, indicating the release of encapsulated DNA via the dissolution of basic LDH nanosheets (lane 3). Under UV–vis illumination, the DNA released from the LDH lattice is completely decomposed (lane 4). The present finding clearly demonstrates that the LDH nanosheet is not adequately stable to serve as a feasible DNA container applicable for diverse DNA-based genetic engineering technologies especially under acidic conditions. In contrast, the DNA–layered titanate nanohybrid possesses very high stability for acidic and UV corrosions, highlighting the reliability of this material as stable nanocontainer for DNA.

**Figure 6:**
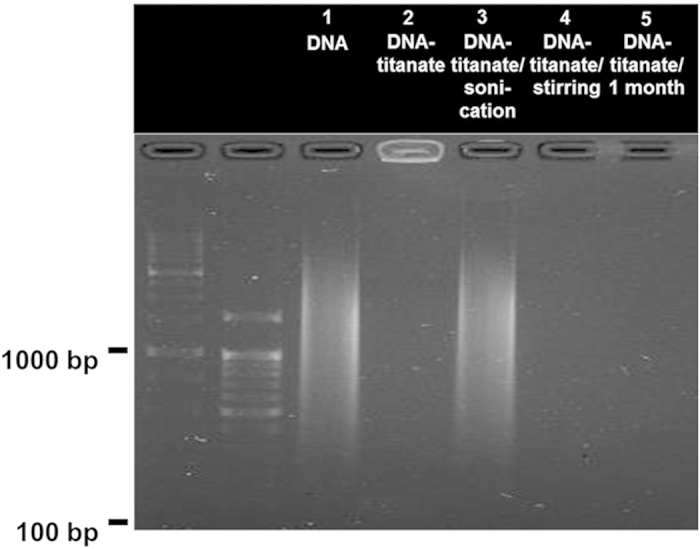
The stability of the DNA−layered titanate nanohybrid against long term storage and recovery test. Agarose gel-electrophoresis analyses of the DNA–layered titanate nanohybrid subjected for long term storage and recovery test. Lane 1: the precursor DNA. Lane 2: the DNA–layered titanate nanohybrid. Lane 3: the DNA–layered titanate nanohybrid after sonication treatment. Lane 4: the DNA–layered titanate nanohybrid after stirring treatment. Lane 5: the acidic solution with pH 5.0 storing DNA–layered titanate nanohybrid under simulated solar irradiation for 1 month. The first and second lanes in the electrophoresis analysis are 1000 and 100 base-pair ladder DNA, respectively. The precursor DNA is used as a positive control.

Additionally the long term-stability of DNA encapsulated in the DNA–layered titanate nanohybrid is monitored in the simulated outdoor conditions of an acidic condition of pH 5.0 (the same pH of acidic rain) and the solar irradiation (AM 1.5; 100 mW cm^−2^). As shown in Fig. 6, no DNA signal appears by agarose gel-electrophoretic analysis for the DNA–layered titanate nanohybrid storing in the acidic media with pH 5.0 for 1 month (lane 5), indicating no release of DNA upon long term storage in the acidic solution under the solar irradiation. The present finding clearly demonstrates the usefulness of the cationic APTES-anchored layered titanate nanosheet as a highly reliable nanocontainer for DNA.

For the practical application of this nanocontainer for DNA technology, the restoration of DNA from the DNA–layered titanate nanohybrid is also crucial. The recovery of DNA from the DNA–layered titanate nanohybrid can be easily achieved by a sonication treatment for 3 minutes. As illustrated in Fig. 6, the release of DNA from the DNA–layered titanate nanohybrid upon the sonication treatment is evidenced by the observation of DNA signal under electric field (lane 3). This result underscores the effective restoration of DNA from the DNA–layered titanate nanohybrid without any notable damage. However, a simple magnetic stirring of the DNA–layered titanate nanohybrid cannot induce the liberation of encapsulated DNA (lane 4), confirming the useful functionality of the present nanohybrid as reliable matrix for the storage of DNA.

### Hybridization of Cationic APTES-Anchored Titanate Nanosheet with Decavanadate Anion

**Figure 7:**
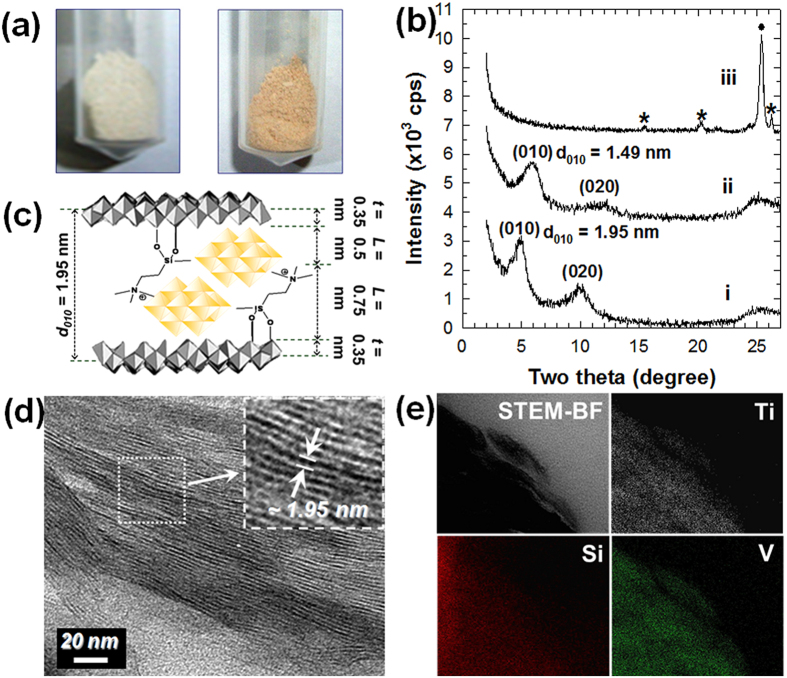
Hybridization of cationic APTES-anchored titanate nanosheet with decavanadate anion. (**a**) Photographs of (left) APTES-anchored layered titanate and (right) the as-prepared decavanadate–layered titanate nanohybrid. (**b**) Powder XRD patterns of i) the as-prepared decavanadate–layered titanate nanohybrid, and its derivatives calcined at ii) 400 °C and iii) 500 °C. (**c**) Structural model, (**d**) cross-sectional HR-TEM image, and (**e**) cross-sectional elemental maps of the as-prepared decavanadate–layered titanate nanohybrid. The asterisks and closed circle in (**b**) indicate Bragg reflections of V_2_O_5_ and anatase TiO_2_ phases, respectively.

In addition to biomolecular anions like DNA, inorganic anionic species like polyoxometalate clusters can also be hybridized with the cationic layered titanate nanosheets by electrostatic attraction. As presented in Fig. 7a, the hybridization with decavanadate nanocluster induces a distinct color change from white to yellow, strongly suggesting the intercalation of decavanadate nanocluster into wide band gap semiconducting titanate. The crystal structure of the decavanadate–layered titanate nanohybrid is examined by powder XRD and HR-TEM analyses. As plotted in Fig. 7b, the decavanadate–layered titanate nanohybrid exhibits a series of equally spaced (*0k0*) reflections with the expanded (010) basal spacing of 1.95 nm, which is larger value than in APTES-anchored layered titanate (d_010_ = 1.67 nm) and indicating the intercalation of decavanadate into the APTES-anchored layered titanate lattice, as illustrated in Fig. 7c. The intercalation of decavanadate nanocluster into the layered titanate lattice is further confirmed by cross-sectional HR-TEM and elemental mapping analyses. As depicted in Fig. 7d, the observation of a series of parallel dark lines with the interlayer distance of ~1.95 nm demonstrates the layer-by-layer ordering of layered titanate nanosheets with decavanadate anions. The cross-sectional electron map in the bright field (BF) of the decavanadate–layered titanate nanohybrid demonstrates the homogeneous distribution of vanadium and silicon elements in the interlayer space of the titanate nanosheets as presented Fig. 7e. According to the scanning transmission electron microscopy (STEM)–energy dispersive spectroscopy (EDS) analysis, the chemical composition of the decavanadate–layered titanate nanohybrid is determined as (V_10_O_28_)_0.064_(APTES)_0.71_Ti_1.83_O_4_ (see Supplementary Information, Fig. S6).

The thermal stability of the decavanadate–layered titanate nanohybrid is examined by monitoring the variation of XRD pattern upon annealing at 400–500 °C in Ar atmosphere. As shown in Fig. 7b, the (0*k*0) reflections of the as-prepared nanohybrid are still observable after the heat-treatment at 400 °C, underscoring the maintenance of the intercalation structure of the as-prepared nanohybrid. The heat treatment at 500 °C suppresses the (0*k*0) reflections of the decavanadate–layered titanate nanohybrid whereas several peaks of V_2_O_5_ and anatase TiO_2_ phases appear, indicating the collapse of the intercalation structure and the phase transformation of the layered titanate to anatase TiO_2_. Lepidocrocite-type layered titanate nanosheet transforms to anatase-type TiO_2_ at 200 °C^8^, thus the observed phase transition occurring at 500 °C can be regarded as clear evidence for the enhancement of the thermal stability of the layered titanate nanosheet upon the formation of strong bonding between cationic APTES-anchored layered titanate nanosheet and decavanadate anion.

The variation of the local structure of vanadium ion in the nanohybrid after the heat treatment at 400–500 °C is also investigated by X-ray absorption near edge structure (XANES) analysis at V K-edge (see Supplementary Information, Fig. S7). The overall spectral features of the decavanadate–layered titanate nanohybrid calcined at 400 °C are similar to the as-prepared decavanadate–layered titanate nanohybrid, indicating the maintenance of the local structure of intercalated decavanadate nanocluster after the heat-treatment at 400 °C. Judging from the phase transition of decavanadate to V_2_O_5_ at 350 °C^39^, the present result demonstrates the enhanced thermal stability of the decavanadate through the immobilization in-between the layered titanate lattice. The nanohybrid calcined at 500 °C displays a significant spectral change to V_2_O_5_-like feature, which is in good agreement with the XRD result showing the phase transformation to V_2_O_5_ at this temperature.

The evolution of the electrode activity of layered titanate upon the hybridization with decavanadate nanocluster is examined with cyclic voltammetry (CV) measurements (see Supplementary Information, Fig. S8). The decavanadate–layered titanate shows significant electrochemical activity, which is sharply contrasted with the negligible activity of the APTES-anchored layered titanate. This result demonstrates the useful role of hybridization with polyoxometalates in improving the electrochemical functionality of metal oxide nanosheet.

In addition to the decavanadate, other polyoxometalate anions such as dichromate ([Cr_2_O_7_]^2−^) and heptamolybdate ([Mo_7_O_24_]^6−^) can be hybridized with the cationic APTES-anchored titanate nanosheet (see Supplementary Information, Fig. S9). The present experimental findings demonstrate the role of the surface-modified layered titanate nanosheet as efficient host materials for inorganic anions.

### CO_2_ Capture by Cationic APTES-Anchored Layered Titanate

**Figure 8:**
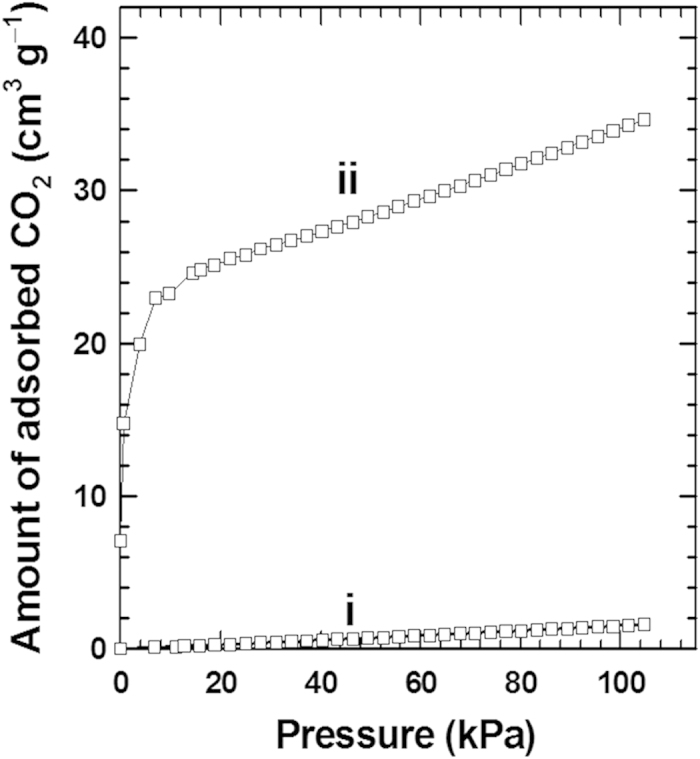
CO_2_ capture by cationic APTES-anchored layered titanate. CO_2_ adsorption behaviors of i) the protonated layered titanate and ii) APTES-anchored layered titanate measured at 273 K up to 1 atm (101.325 kPa).

Taking into account the basic nature of the APTES groups, the APTES-anchored titanate is also tested as CO_2_ adsorbent. As illustrated in Fig. 8, this material shows CO_2_ adsorption ability of 33.6 cm^3^ g^−1^ (i.e. 1.55 mmolg^−1^) at STP. There are several reports regarding the CO_2_ adsorption abilities of MOF compounds measured at 273 K and 1 atm (STP), e.g. [(Ni_2_L^4^)(bptc)]: ~0 cm^3^ g^−1^ at STP; [Co^II^_4_(μ-OH_2_)_4_(MTB)_2_]_n_: 35.7 cm^3^ g^−1^ at STP; [Zn_3_-(ntb)_2_]_n_: 35.3 cm^3^ g^−1^ at STP^40^,^41^,^42^. The CO_2_ adsorption abilities of these MOF compounds are comparable to the APTES-anchored layered titanate. This result indicates that the APTES-anchored titanate nanosheet can be used as an efficient CO_2_ adsorbent. The strong CO_2_ adsorption ability of APTES-anchored layered titanate is attributed to the high reactivity between the amine group of APTES and the CO_2_ to form carbamate. This reaction, known as Dankwerts’ zwitterion mechanism, can be expressed as follows^43^.


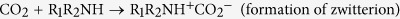



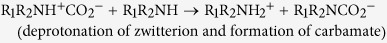


This result highlights that the surface modification of layered metal oxide with basic APTES groups provides an effective way to explore novel efficient CO_2_ adsorbents and demonstrates another merit of 2D morphology.

## Discussion

A new universal platform of cationic metal oxide nanosheet for the hybridization of diverse anionic species was developed by the anchoring of APTES molecules on the surface of layered titanate. The anchoring of amine group makes possible the conversion of the negative surface charge of layered metal oxide nanosheet to positive, yielding a novel cationic metal oxide 2D nanosheet. Diverse anionic species including DNA can be hybridized with the resulting APTES-anchored layered titanate nanosheet via electrostatically-derived assembly. This cationic APTES-anchored titanate nanosheet can act as a highly stable nanocontainer for reliable protection and restoration of DNA. The encapsulation of DNA in the cationic titanate nanosheet remarkably enhances its stability against various corrosions such as enzymatic cleavage of DNA, acidic etching, UV−vis irradiation, and long term-storage. The present DNA−layered titanate nanohybrid with specific genetic manipulation/information is useful in developing new genetic coding system or sensors with bio-molecules. This new technology would provide much higher precision and better confidentiality than any other identification methods such as barcodes, watermarkers, and tag^14^,^44^,^45^. In comparison with an LDH nanosheet previously used for the hybridization with DNA^27^,^32^, the present cationic APTES-anchored layered metal oxide nanosheet has inherently high stability for enzymatic cleavage, acidic corrosion, and high temperature. Besides the application for DNA nanocontainer, the cationic APTES-anchored layered titanate nanosheet can act as novel host materials for diverse anionic species like vanadate, chromate, and molybdate, and also as efficient CO_2_ adsorbent. The application of the present surface-modified metal oxide nanosheet as a building block provides a novel synthetic route to many novel nanohybrids composed of layered metal oxide and anionic nanoclusters with unprecedented chemical compositions and functionalities.

## Methods

### Synthesis of Cationic APTES-Anchored Titanate Nanosheet

Layered cesium titanate, Cs_0.67_Ti_1.83_O_4_, with a lepidocrocite-type structure was prepared by solid state reaction^25^. The exchange of interlayer cesium ions with protons was achieved by vigorous stirring of the HCl suspension containing the pristine Cs_0.67_Ti_1.83_O_4_ powder^25^. The octylamine-intercalated layered titanate was obtained by vigorous stirring of the aqueous suspension of protonated titanate with octylamine for 120 h. For the anchoring of APTES on the surface of titanate, the octylamine-intercalated layered titanate was reacted with APTES in toluene media at 60 °C for 48 h with N_2_ bubbling. The resulting APTES-anchored layered titanate was washed thoroughly with acetone and vacuum-dried at 80 °C. The exfoliation into the cationic layered titanate nanosheet was achieved by the ultrasonication of the aqueous suspension of the APTES-anchored layered titanate for 15 min at fixed pH 4.0.

### Hybridization with DNA

DNA from herring testes (Sigma-Aldrich, D6898, type XIV) was purified with a phenol/chloroform/isoamylalcohol solution and sheared off to the size of 50–5000 base pairs, according to the conventional method^27^,^46^. The DNA solution (3 times excess) was added dropwise to the colloidal suspension of APTES-anchored layered titanate nanosheets. An electrostatic attraction between two oppositely charged species led to the immediate flocculation of two kinds of colloidal particles. The pH of the reactant solution was adjusted to 4.0. The hybridization between DNA and APTES-anchored layered titanate was accomplished by gentle stirring at ambient temperature for 48 h. The resulting DNA−layered titanate nanohybrid was thoroughly washed with deionized water and dried at 60 °C.

### Restoration of Encapsulated DNA

To restore the encapsulated DNA from the layered titanate lattice, an aqueous suspension of the DNA−layered titanate material was treated with sonication for 3 min. Excess NaCl salt with 5 M was used as charge-stabilizer to prevent from reassembling of these two components. After the sonication, the DNA−layered titanate powder was separated from the solution by centrifugation to isolate the released DNA.

### Hybridization with Polyoxometalate Anions

For the hybridization between decavanadate ([V_10_O_28_]^6−^) anions and the APTES-anchored layered titanate, an aqueous solution containing excess amount of ammonium metavanadate was added dropwise to the colloidal suspension of the APTES-anchored layered titanate nanosheets. The mole ratio of V:Ti was fixed to 3:1. The pH of the reactant solution was adjusted to pH 4.5 with 0.1 M HCl solution for the predominant formation of decavanadate cluster^20^. Flocculation occurred immediately after the addition of anionic species to the colloidal suspension of APTES-anchored layered titanate nanosheets, a result of the electrostatic interaction between two oppositely charged species. The reaction proceeded for 72 h in ambient atmosphere. The resulting material was washed with deionized water and dried at 60 °C. The universal applicability of the APTES-anchored layered titanate nanosheet as a host material was tested for various polyoxometalate anions. Instead of ammonium metavanadate, the precursor of sodium chromate or sodium molybdate dihydrate was utilized for the synthesis of dichromate−layered titanate or heptamolybdate−layered titanate nanohybrid, respectively^20^.

### Stability Tests of DNA−Layered Titanate Nanohybrid

The stability of the DNA−layered titanate nanohybrid against enzymatic corrosion was examined by storing the material in the solution of DNase I (Sigma-Aldrich, D4263) for 2 h^27^. The effects of acidic and UV−vis light corrosions on the stability of the DNA−layered titanate nanohybrid were investigated by soaking the material in acidic solution of pH 2 for 24 h and then irradiating with UV−vis light for 72 h. As a reference for the test of acidic corrosion, DNA−LDH (Mg_2_Al(OH)_6_) nanohybrid was also synthesized as previously reported^35^. Additionally the long term stability of the DNA−layered titanate nanohybrid was tested by storing the material in acidic solution of pH 5.0 for 1 month under UV−vis illumination. After all the stability tests, the DNA encapsulated in the nanohybrid material was examined with agarose gel-electrophoresis analysis.

### Electrochemical Measurements of Decavanadate−Layered Titanate Nanohybrid

The electrode functionality of the decavanadate−layered titanate nanohybrid was examined by the CV analysis. The composite electrode was prepared with the mixture slurry of the active material (80wt%), Super P (10wt%), and PVDF (10wt%) in N-methyl-2-pyrrolidene (NMP). The obtained slurry was deposited on copper foil and dried in vacuum oven at 120 °C for 12 h. The electrochemical cycling tests were carried out with the cell of Li/1M LiPF_6_ in ethylene carbonate (EC): diethyl carbonate (DEC) (50:50 in vol%; 97vol%) and fluoroethylene carbonate (FEC) (3vol%)/active material, which was assembled in a dry-box. The CV data were collected with potentiostat in the voltage range of 0.1–3.0 V with scan rate of 10 mVs^−1^.

### Measurement of CO_2_ Adsorption Capacity

The CO_2_ adsorption capacity of the APTES-anchored layered titanate was measured at 273 K up to 1 atm using a static volumetric apparatus (Micrometrics, ASAP 2020 adsorption analyzer).

### Characterization

The crystal structures and the natures of the chemical bonding of the present nanohybrids were examined by powder XRD analysis and FT-IR spectroscopy, respectively. To probe the formation of siloxane bonds between APTES and layered titanate, solid-state ^29^Si MAS-NMR spectrum of the APTES-anchored layered titanate was collected with a Bruker DSX 400 NMR spectrometer at the Korea Basic Science Institute operating at 79.49 MHz using a cross-polarization−MAS probe and a 5 mm ZrO_2_ rotor. The chemical compositions of octylamine-intercalated layered titanate and APTES-anchored layered titanate were estimated with TG analysis, AES−ICP spectrometry, and elemental CHN analysis. The pH-dependent dispersion abilities of the present materials were examined by dispersing the corresponding powders in deionized water at variable pH conditions. The evolution of the surface charge of layered titanate upon the anchoring of APTES was monitored by measuring zeta potential (zetasizer Nano ZS, Malvern Instruments). The thickness and crystallite dimension of the exfoliated APTES-anchored layered titanate nanosheets were determined with AFM (PSIA, XE-100) analysis in a tapping-mode. The stacking structures, crystal morphologies, and chemical compositions of the present nanohybrids were examined using HR-TEM and FE-SEM with EDS, respectively. The elemental composition of the decavanadate−layered titanate nanohybrid was probed using STEM with elemental mapping and EDS analyses. The nature of the chemical bonding of the decavanadate−layered titanate nanohybrid was studied with XANES analysis at V K-edge.

## Additional Information

**How to cite this article**: Kim, T. W. *et al*. Highly Stable Nanocontainer of APTES-Anchored Layered Titanate Nanosheet for Reliable Protection/Recovery of Nucleic Acid. *Sci. Rep*. **6**, 21993; doi: 10.1038/srep21993 (2016).

## Supplementary Material

Supplementary Information
