# Supplementary material for: Highly Stable Nanocontainer of APTES-Anchored Layered Titanate Nanosheet for Reliable Protection/Recovery of Nucleic Acid
Source: Sci Rep. 2016 Feb 24;6:21993. doi: 10.1038/srep21993 (PMC4764942; doi:10.1038/srep21993)
Supplement: Supplementary Information [file srep21993-s1.pdf]

Supplementary Information

Highly Stable Nanocontainer of APTES-Anchored Layered Titanate Nanosheet for Reliable Protection/Recovery of Nucleic Acid

Tae Woo Kim[1,+], In Young Kim[1,+], Dae-Hwan Park[1], Jin-Ho Choy[1], and Seong-Ju Hwang[1,*]

[1] Department of Chemistry and Nanoscience, Ewha Womans University, Seoul 03760, Korea

* To whom all correspondences should be addressed.

[+] These authors contributed equally to this work.

Tel) +82-2-3277-4370

Fax) +82-2-3277-3419

E-mail) hwangsju@ewha.ac.kr

Details of Characterization

The crystal structures of the obtained titanate-based materials were examined by powder X-ray diffraction (XRD) analysis (Rigaku, λ = 1.5418 Å, 298 K, Ni-filtered Cu K□-radiation). The chemical bonding nature of the present nanohybrids was examined with Fourier transformed-infrared (FT-IR) spectroscopy with Varian SPS-800 spectrometer employing the KBr dilution technique. Solid-state ^{29}Si magic angle spinning-nuclear magnetic resonance (MAS-NMR) spectrum was collected with a Bruker DSX 400 NMR spectrometer at the Korea Basic Science Institute operating at 79.49 MHz using a cross polarization MAS probe and a 5 mm ZrO_2 rotor. MAS experiment was performed at 4.5 kHz. Data acquisition was carried out via a single contact Hartman Hahn Cross-Polarization pulse sequence. The ^1H 90° pulse length was 5 μs, and the recycle time was 3 s. The cross polarization contact time was 20 ms. The ^{29}Si NMR chemical shifts recorded on the δ-scale were referenced through external tetrakistrimethylsilane. The chemical compositions and thermal behaviors of octylamine-intercalated layered titanate and (3-aminopropyl)triethoxysilane molecules (APTES)-anchored layered titanate were estimated by performing induced coupled plasma (ICP) spectrometry (Shimazu ICPS-5000), elemental CHN analysis (CE-Instruments-EA-1110), and thermogravimetric (TG, Rigaku TAS-100) analysis, respectively. Dispersion tests were done by dispersing APTES-anchored layered titanate, octylamine-intercalated layered titanate, and unmodified titanate in deionized water at several pH conditions. The thickness and crystallite dimension of the APTES-anchored layered titanate nanosheets were determined with atomic force microscopy (AFM, PSIA, XE-100) analysis in a tapping-mode. The APTES-anchored layered titanate nanosheets were adsorbed onto a Si wafer substrate. A zeta potential of the colloidal suspension of APTES-anchored layered titanate was measured as a function of pH in the range from 2 to 10, with the zetasizer Nano ZS (Malvern Instruments). The pH of the suspension was adjusted using 0.01 M aqueous HCl or NaOH solution through the autotitration set-up. The diluted suspension of APTES-anchored layered titanate was kept at 25 °C and started to circulate into the zeta cell. After stabilization from circulation for ~3–5 min, the zeta potential of the colloidal suspension of APTES-anchored layered titanate nanosheet was recorded. For comparison, the zeta potential of unmodified titanate colloid was also measured. The crystal morphology of the DNA–layered titanate nanohybrid was examined with field emission-scanning electron microscopy (FE-SEM) using a JEOL JSM-6700F microscope. The chemical compositions of the decavanadate–layered titanate and DNA–layered titanate nanohybrids were determined with a JEOL JSM-6700F microscope equipped with an energy dispersive X-ray spectrometer (EDS). The stacking structure and elemental distribution of the decavanadate–layered titanate were examined using high resolution-transmission electron microscopy (HR-TEM, JEOL JEM-2100F microscope, 200 kV) with EDS–elemental mapping analysis. Agarose gel-electrophoresis analysis was carried out to confirm the incorporation of DNA in the lattice of the DNA–layered titanate nanohybrid. After the sample was loaded on a 1% agarose gel, an electrical potential of 100 V was applied for the sample for 30 min. After the operation, the agarose gel was stained with 100 μgL^{-1} ethidium bromide (EtBr) for DNA visualization and was imaged under UV illumination. To probe the effect of hybridization on the stability of DNA, the stability of the DNA–layered titanate nanohybrid, precursor DNA used in this study, and DNA intercalated in layered double hydroxide (LDH) were tested under two conditions as follows. First, each required sample was treated with acidic solution of low pH = 2 for one day under stirring. Then, UV–vis radiation was illuminated for 72 h on each sample in acidic solution. For this experiment, a 450 W Xe arc lamp (Oriel) was used as light-source. After the stability tests, the chemical state of DNA molecules in the samples was probed with agarose gel

electrophoresis. In order to restore the DNA molecules from APTES-anchored layered titanate layer, an aqueous solution containing the powdery DNA–layered titanate material was treated by using a sonicator for less than 3 min. Excess NaCl salt with 5 M was used as charge-stabilizer to protect reassembly of separated two materials. After the sonication, the DNA–layered titanate powder in the solution was removed by centrifugation. To obtain cross-sectional images of the heterostructure, the decavanadate–layered titanate sample was blended with acrylic resin (4:6 methyl methacrylate:n-butyl methacrylate, 1.5% benzoyl peroxide) in a polyethylene capsule, and then the sample was sliced using an ultramicrotome with a diamond knife. X-ray absorption near-edge structure (XANES) spectra at V K-edge were collected from the thin layer of powder samples deposited on transparent adhesive tapes in a transmission mode at beam line 10C of Pohang Light Sources (Pohang, Korea) operated at 2.5 GeV and 180 mA. The measurements were carried out at room temperature with a Si (111) single-crystal monochromator. All of the present XANES spectra were calibrated by measuring the spectrum of vanadium or titanium metal foil. CO_2 adsorption capacities of the protonated layered titanate and APTES-anchored layered titanate were measured at 273 K up to 1 atm (101.325 kPa) using a static volumetric apparatus (ASAP 2020 adsorption analyzer). Prior to measurements, all the samples were degassed at 150 °C for 5 h under vacuum.

Supplementary Figure S1. (Left) Powder XRD patterns of (a) layered cesium titanate, (b) protonated titanate, (c) octylamine-intercalated layered titanate, and (c) APTES-anchored layered titanate. (Right) Structural models of the present titanate materials.

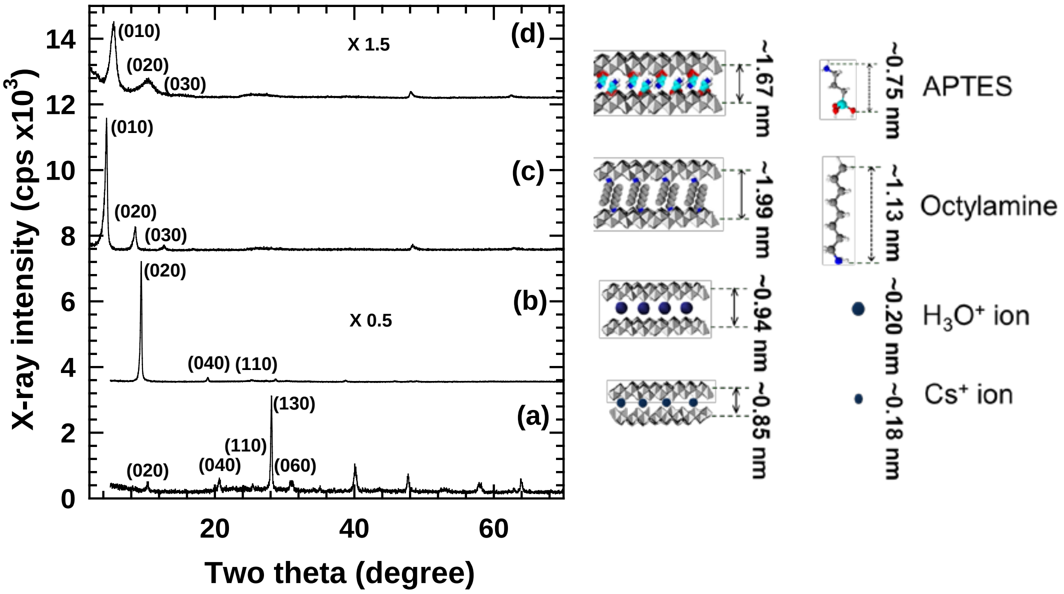

: Evolutions of the crystal structure of layered titanate upon octylamine intercalation and APTES anchoring are investigated with powder XRD analysis. As shown in the left panel of **Figure S1**, the octylamine-intercalated layered titanate shows a series of equally spaced (*0k0*) Bragg reflections in lower angle region than does the protonated layered titanate, indicating the intercalation of long chain octylamine molecule. After the anchoring of APTES, the (*0k0*) reflections are displaced toward higher angle side, suggesting the decrease of the basal spacing. According to the least-squares fitting analysis, the expanded basal spacing of octylamine-intercalated layered titanate (1.99 nm) is notably decreased to 1.67 nm for the APTES-intercalated layered titanate, underscoring the replacement of longer octylamine molecules (theoretical length: ~1.13 nm) with shorter APTES ones (theoretical length: ~0.75 nm), as illustrated in right panel of Figure S1.

Supplementary Figure S2. FT-IR spectra of (a) layered cesium titanate, (b) octylamine-intercalated layered titanate, and (c) APTES-anchored layered titanate.

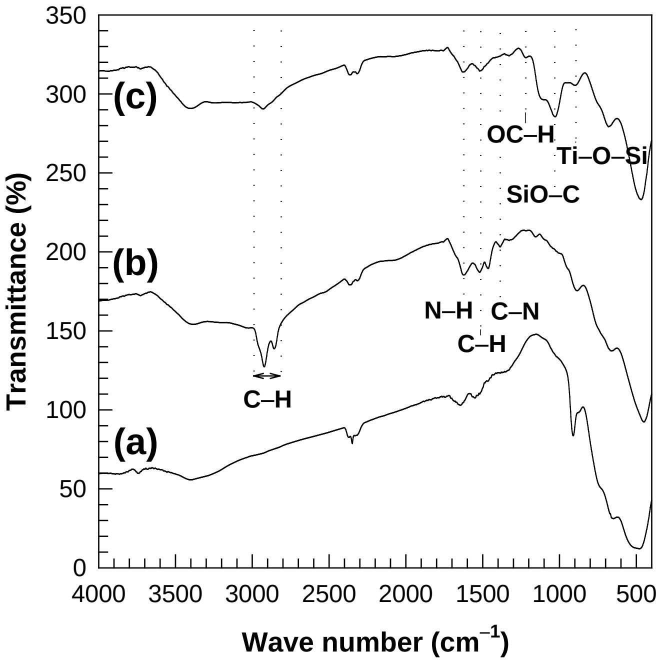

: The FT-IR spectra of the layered cesium titanate, octylamine-intercalated layered titanate, and APTES-anchored layered titanate are presented in **Figure S2**. The octylamine-intercalated layered titanate displays C–H stretching bands at 2957, 2922, and 2852 cm^{-1}, C–H bending vibration band at 1468 cm^{-1}, C–N stretching vibration band at 1370 cm^{-1}, and N–H bending vibration at 1640 cm^{-1}. The present finding clearly demonstrates the intercalation of octylamine in-between the titanate layers. The IR spectrum of the APTES-anchored layered titanate shows characteristic absorption bands corresponding to APTES-silyl groups;[1] OC–H bending vibration band at ~1212 cm^{-1} and stretching vibration bands of SiO–C at ~990–1100 cm^{-1} [2,3]. Additionally, the IR band spectrum of the APTES-anchored layered titanate displays N–H bending vibration band of amine at 1640 cm^{-1}. Of prime importance is that an absorption band corresponding to Si–O–Ti bonds is discernible at ~970 cm^{-1} [2,3], underscoring the anchoring of APTES molecules on the interlayer surface of layered titanate. Additionally the APTES-anchored layered titanate exhibits strong absorption bands related to TiO_6 octahedra at below 930 cm^{-1}, confirming the maintenance of layered titanate lattice upon the APTES anchoring.

(1) Smith, A. L. *Spectrochim. Acta* **16**, 87 (1960).

(2) Ide, Y. & Ogawa, M. *J. Colloid Interf. Sci.* **296**, 141 (2006).

(3) Ide, Y. & Ogawa, M. *Angew. Chem.* **46**, 8449 (2007).

Supplementary Figure S3. (Left) Transmission electron microscopic (TEM) images of the DNA–layered titanate nanohybrid and (right) selected area electron diffraction (SAED) pattern obtained from the edge of the DNA–layered titanate nanohybrid.

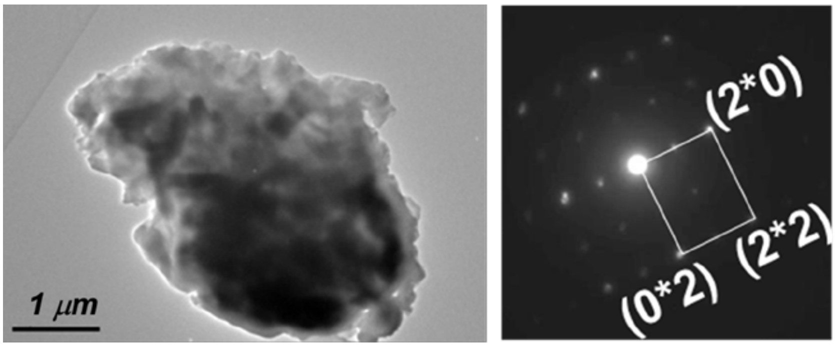

: As illustrated in the left panel of **Figure S3**, the DNA–layered titanate nanohybrid displays irregular spherical particles whose surface is wrapped by cationic titanate nanosheets, confirming the encapsulation of anionic DNA molecules in the titanate lattice. The observation of the in-plane diffraction patterns of (2*0) and (0*2) from SAED data confirms the maintenance of the 2D lattice of the titanate nanosheets after the hybridization with DNA, see the right panel of Figure S3.

Supplementary Figure S4. (Left) FE-SEM image, (middle) EDS spectrum, and (right) elemental contents of the DNA–layered titanate nanohybrid.

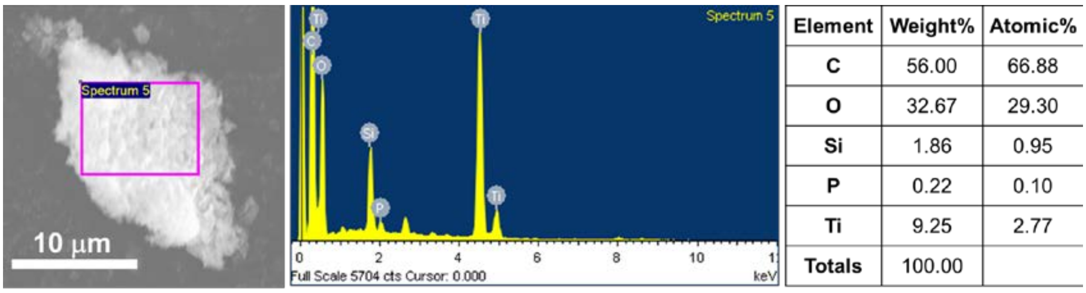

Element	Weight%	Atomic%
C	56.00	66.88
O	32.67	29.30
Si	1.86	0.95
P	0.22	0.10
Ti	9.25	2.77
Totals	100.00	

: As illustrated in **Figure S4**, the DNA content in the present DNA–layered titanate nanohybrid is examined with EDS analysis, indicating the Ti:P atomic ratio of 2.77:0.1. The DNA content in the nanohybrid can be calculated as follows. Since one DNA molecule has one phosphorous element, the EDS result presented here indicates that the DNA–layered titanate nanohybrid possesses a chemical composition of $(APTES)_{0.71}Ti_{1.83}O_4{\cdot}0.8H_2O{\cdot}0.066DNA$. On the basis of the molecular weights of $(APTES)_{0.71}Ti_{1.83}O_4{\cdot}0.8H_2O$ (323.19) and DNA (499.45), the mass percent of DNA in the present nanohybrid of $(APTES)_{0.71}Ti_{1.83}O_4{\cdot}0.8H_2O{\cdot}0.066DNA$ can be calculated to be 9.3wt%. This result implies that the present DNA–layered titanate nanohybrid can store ~93 mg of DNA per 1 g of the nanohybrid.

Supplementary Figure S5. (Left) UV–vis spectra of the precursor DNA and the APTES-anchored layered titanate nanosheet and (right) an illustration for protection of DNA by the APTES-anchored layered titanate from UV–vis light.

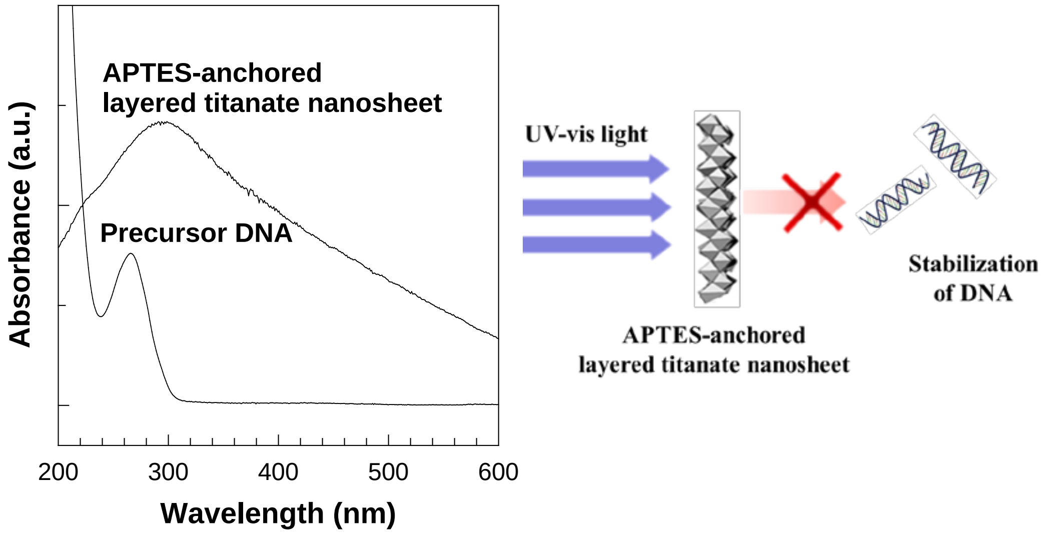

: As can be seen clearly from the left panel of **Figure S5**, the precursor DNA molecule shows a typical absorption peak at 266 nm in the region of UV light, whereas the APTES-anchored layered titanate nanosheet can absorb UV light at 260–350 nm. Since the outer shell of the APTES-anchored layered titanate can firstly absorb an incident UV light, the UV-induced photodegradation of DNA becomes effectively supressed upon the encapsulation in the titanate lattice.

Supplementary Figure S6. (Left) Cross sectional scanning transmission electron microscopy–bright field (STEM–BF) image, (middle) EDS spectrum, and (right) elemental contents of the decavanadate–layered titanate nanohybrid.

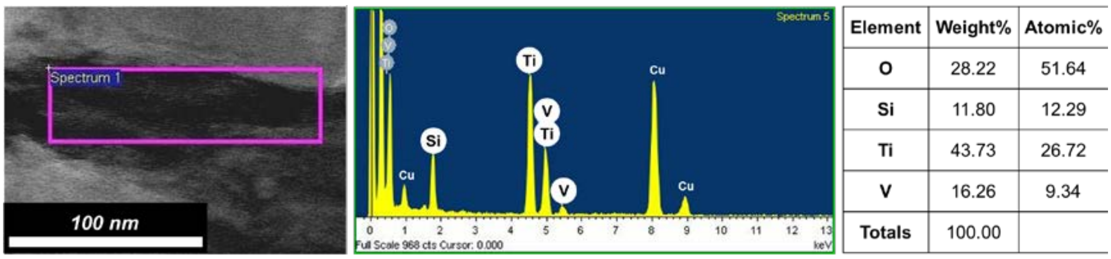

Element	Weight%	Atomic%
O	28.22	51.64
Si	11.80	12.29
Ti	43.73	26.72
V	16.26	9.34
Totals	100.00	

: As illustrated in **Figure S6**, the chemical composition of the decavanadate–layered titanate nanohybrid is examined with EDS analysis, showing the Ti:Si:V atomic ratio of 26.72:12.29:9.34. On the basis of this result, the chemical composition of the decavanadate–layered titanate nanohybrid is determined as $(V_{10}O_{28})_{0.064}(APTES)_{0.71}Ti_{1.83}O_4$. The present decavanadate–layered titanate nanohybrid can accommodate ~165 mg of decavanadate per 1 g of the nanohybrid.

Supplementary Figure S7. V K-edge XANES spectra of (a) the as-prepared decavanadate–layered titanate nanohybrid, its derivatives calcined at (b) 400 and (c) 500 °C, and (d) V_2O_5.

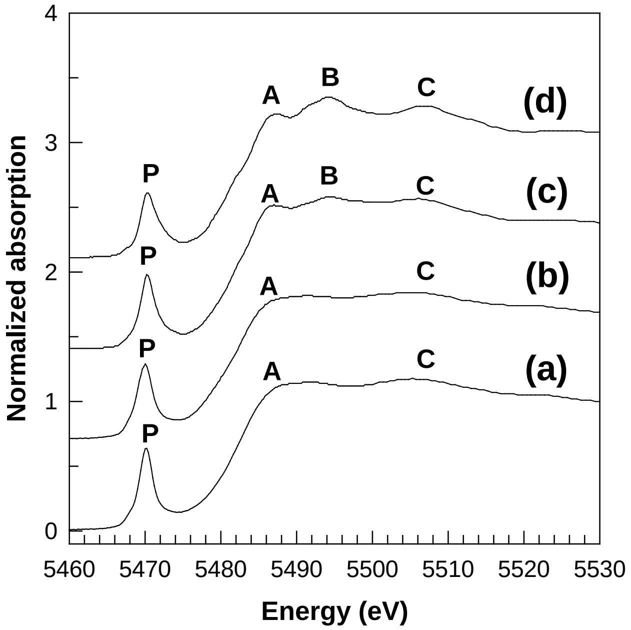

: The local structure of vanadium ion is investigated with V K-edge XANES analysis. As shown in **Figure S7**, the overall spectral feature of the decavanadate–layered titanate nanohybrid calcined at 400 °C is similar to that of the as-prepared nanohybrid, underscoring the maintenance of decavanadate nanocluster upon the heat treatment at 400 °C. Conversely, the calcination at 500 °C leads to a significant change of the V K-edge XANES feature of the as-prepared nanohybrid to that of the reference V_2O_5, confirming the phase transformation into vanadium pentoxide.

Supplementary Figure S8. Cyclic voltammetry (CV) curves of (left) the APTES-anchored layered titanate and (right) the as-prepared decavanadate–layered titanate nanohybrid for the first (red), second (blue), and third cycle (black).

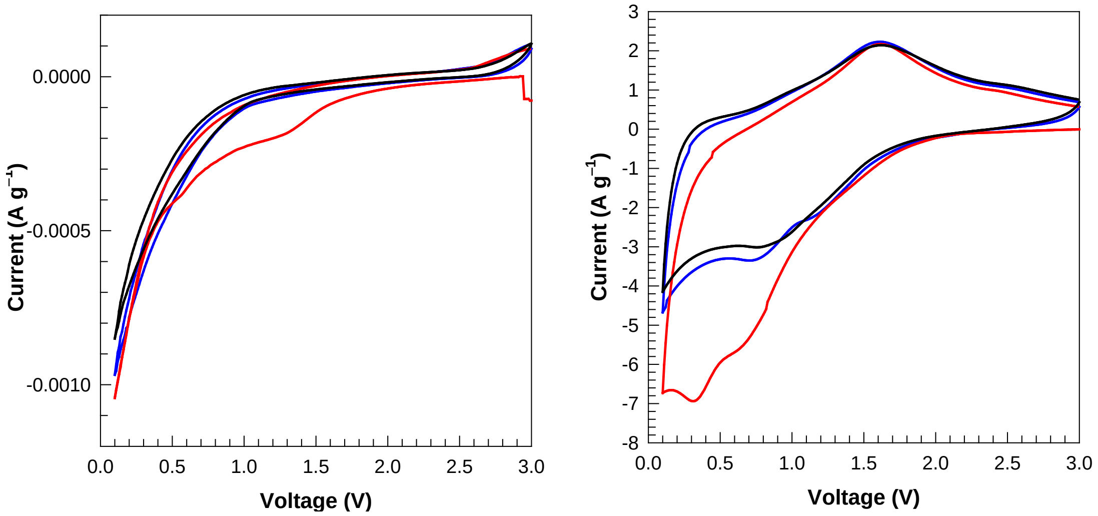

: The evolution of the electrochemical activity of layered titanate upon the hybridization with decavanadate is examined with CV measurement. As illustrated **Figure S8**, the decavanadate–layered titanate shows much higher electrochemical activity than does the APTES-anchored layered titanate. This result clearly demonstrates the useful role of the hybridization with polyoxometalates in enhancing the electrode functionality of layered metal oxide nanosheet.

Supplementary Figure S9. Photographs of a series of new nanohybrids composed of the APTES-anchored layered titanate and several polyoxometalate nanoclusters such as decavanadate, dichromate, and heptamolybdate.

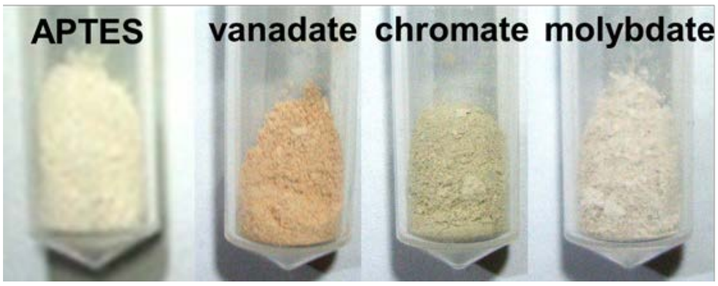

: In terms of an electrostatic interaction, the positively-charged APTES-anchored layered titanate nanosheet can be hybridized with anionic guest species like polyoxometalate clusters. As evidenced from the color change in **Figure S9**, various anionic polyoxometalates like decavanadate, dichromate, and heptamolybdate can be hybridized with APTES-anchored layered titanate nanosheet. The present result provides clear evidence for the universal role of the present cationic layered titanate as host material for various anionic guest species.
